# Requirement Analysis for Data-Driven Electroencephalography Seizure Monitoring Software to Enhance Quality and Decision Making in Digital Care Pathways for Epilepsy: A Feasibility Study from the Perspectives of Health Care Professionals

**DOI:** 10.2196/59558

**Published:** 2025-05-30

**Authors:** Pantea Keikhosrokiani, Johanna Annunen, Jonna Komulainen-Ebrahim, Jukka Kortelainen, Mika Kallio, Päivi Vieira, Minna Isomursu, Johanna Uusimaa

**Affiliations:** 1 Empirical Software Engineering in Software Systems and Services Faculty of Information Technology and Electrical Engineering University of Oulu Oulu Finland; 2 Research Unit of Health Sciences and Technology Faculty of Medicine University of Oulu Oulu Finland; 3 University of Oulu Oulu Finland; 4 Oulu University Hospital Medical Research Center Neurocenter (Member of ERN EpiCARE) Oulu Finland; 5 Oulu University Hospital Oulu Finland; 6 Center for Machine Vision and Signal Analysis Faculty of Information Technology and Electrical Engineering University of Oulu Oulu Finland; 7 Cerenion Ltd Oulu Finland; 8 Research Unit of Clinical Medicine Faculty of Medicine University of Oulu Oulu Finland

**Keywords:** ubiquitous health, uHealth, data-driven software, requirement analysis, epilepsy seizure monitoring, digital care pathway, electroencephalography, Delphi study, focus group, artificial intelligence, AI

## Abstract

**Background:**

Abnormal brain activity is the source of epileptic seizures, which can present a variety of symptoms and influence patients’ quality of life. Therefore, it is critical to track epileptic seizures, diagnose them, and provide potential therapies to manage people with epilepsy. Electroencephalography (EEG) is helpful in the diagnosis and classification of the seizure type, epilepsy, or epilepsy syndrome. Ictal EEG is rarely recorded, whereas interictal EEG is more often recorded, and the results can be abnormal or normal even in the case of epilepsy. The current digital care pathway for epilepsy (DCPE) lacks the integration of data-driven seizure detection, which could potentially enhance epilepsy treatment and management.

**Objective:**

This study aimed to determine the requirements for integrating data-driven medical software into the DCPE to meet the project’s goals and demonstrate practical feasibility regarding resource availability, time constraints, and technological capabilities. This adjustment emphasized ensuring that the proposed system is realistic and achievable. Perspectives on the feasibility of data-driven medical software that meets the project’s goals and demonstrates practical feasibility regarding resource availability, time constraints, and technological capabilities are presented.

**Methods:**

A 4-round Delphi study using focus group discussions was conducted with 7 diverse panels of experts from Oulu University Hospital to address the research questions and evaluate the feasibility of data-driven medical software for monitoring individuals with epilepsy. This collaborative approach fostered a thorough understanding of the topic and considered the perspectives of various stakeholders. In addition, a qualitative study was carried out using semistructured interviews.

**Results:**

Drawing from the findings of the thematic analytics, a detailed set of guidelines was created to facilitate the seamless integration of the proposed data-driven medical software for EEG seizure monitoring into the DCPE. These guidelines encompass system requirements, data collection and analysis, and user training, offering a comprehensive road map for the effective implementation of the software.

**Conclusions:**

The study outcome presents a comprehensive strategy for improving the quality of care, providing personalized solutions, managing health care resources, and using artificial intelligence and sensor technology in clinical settings. The potential of artificial intelligence and sensor technology to revolutionize health care is exciting. The study identified practical strategies, such as real-time EEG seizure monitoring, predictive modeling for seizure occurrence, and data-driven analytics integration to enhance decision-making. These strategies were aimed at reducing diagnostic delays and providing personalized care. We are actively working on integrating these features into clinical workflows. However, further case studies and pilot implementations are planned for future studies. The results of this study will guide system developers in the meticulous design and development of systems that meet user needs in the DCPE.

## Introduction

### Background

Epilepsy is a neurological condition that is characterized by recurrent and unprovoked seizures [1]. Epilepsy is one of the most common neurological conditions, affecting millions of people worldwide [2]. Epileptic seizures occur due to abnormal brain electrical activity, leading to a wide variety of symptoms and manifestations. This condition can substantially impact patients’ quality of life. Therefore, to effectively manage and provide support for people with epilepsy, it is essential to understand epilepsy, including its causes, diagnosis, available treatments, and comorbidities [3]. One of the most critical aspects of adequate care is knowing thoroughly about the seizure types, etiology, and provoking factors [4,5].

Electroencephalography (EEG) data play a crucial role in detecting epileptic seizures by capturing distinct patterns and anomalies associated with epileptic seizures by recording the brain’s electrical activity. Sharp waves and spikes, slow waves, rhythmic patterns, and interictal epileptiform discharges are some of the possible EEG findings of people with epilepsy [4,6-10]. Experienced neurologists or automated algorithms can visually identify and analyze these abnormal patterns. The possibilities of EEG data processing for seizure detection have been substantially enhanced by recent developments in machine learning and artificial intelligence (AI) [11,12]. It is possible to train algorithms to recognize and categorize seizure occurrences automatically, giving individuals or health care professionals real-time notifications.

On the basis of the discussion with health care professionals, the current digital care pathway for epilepsy (DCPE) faces several issues and shortcomings. Despite therapeutic advances, the classification, diagnosis, and treatment of epilepsy remain complex. Current medications only manage seizures in around two-thirds of people with epilepsy, which emphasizes the need for more potent therapies [13]. The current DCPE lacks data-driven seizure detection integration, which could enhance differential diagnosis and treatment in drug-resistant patients. The addition of data-driven technologies could offer significant improvements, including more accurate seizure detection and prediction, personalized treatment plans, better management of medication-resistant epilepsy, and shared decision-making. However, implementing AI solutions such as machine learning for seizure detection and monitoring is not yet a standard component of DCPE. Integrating a new data-driven medical software for EEG seizure monitoring into the current DCPE requires a thorough understanding of the user’s needs and system requirements. Furthermore, robust requirement engineering and precise stakeholder requirements definitions are needed to design and develop data-driven medical software to ensure patient safety, privacy, compliance with regulatory standards, functional accuracy and relevance, interpretability, scalability, and sustainability [14-16].

### This Study

This study aimed to define the system requirements for developing and integrating data-driven medical software for EEG seizure monitoring (EEG-EpiDigi) in a clinical setting. The system requirement analysis (SRA) focused on assessing user needs, gathering those requirements, analyzing them, and prioritizing their importance. While patient-centered care is a key focus of this system, the initial phase of the study emphasized collecting insights from health care professionals about system requirements. Future research will include patient perspectives and experiences, particularly regarding home monitoring systems, to ensure the software is user friendly. This study explicitly investigated health care professionals’ opinions to identify the requirements for implementing data-driven medical software for EEG seizure monitoring within hospitals, thereby assisting in their decision-making processes. The main research questions of this study were as follows:

- What are the user needs from EEG-EpiDigi in DCPE?
- What are the system requirements for developing and integrating the EEG-EpiDigi system in the current DCPE?
- What are the priorities of the EEG-EpiDigi system in the current DCPE?

The study aimed to determine the feasibility of integrating data-driven medical software in the DCPE. After addressing the research questions and assessing the proposed system requirements, a 4-round Delphi study using focus group discussions with health care professionals from Oulu University Hospital, including neurologists, digital health specialists, pediatric neurologists, experts in EEG signal processing, and clinical neurophysiologists, was conducted from May to November 2023. Semistructured interviews were conducted, and the data were analyzed qualitatively. The final round of the Delphi study directs the discussion to the system requirements confirmation as the first step of the new EEG-EpiDigi system development life cycle. The remainder of the paper is organized as follows: the details of the proposed methodology, such as study design, participants, interview guide, and data analysis, are discussed. Afterward, the results and analysis are added, which include the data exploration, study mind map, and the thematic analysis of the system requirements for EEG-EpiDigi. The paper is wrapped up with a Discussion section, including the futuristic architecture for EEG-EpiDigi and the proposed SRA-EpiDigi.

## Methods

### Overview

This study focused on expert opinions to determine the requirements of implementing and integrating data-driven medical software for EEG seizure monitoring in the current DCPE within hospitals and to assist health care professionals in decision-making. Although the proposed system emphasizes patient-centered solutions, the primary goal of this initial research was to collect only health care professionals’ opinions on the system’s needs. This study was done to implement the proposed system within the hospital only, whereas future research focuses more on home monitoring systems to take patient viewpoints into account.

**Figure 1:**
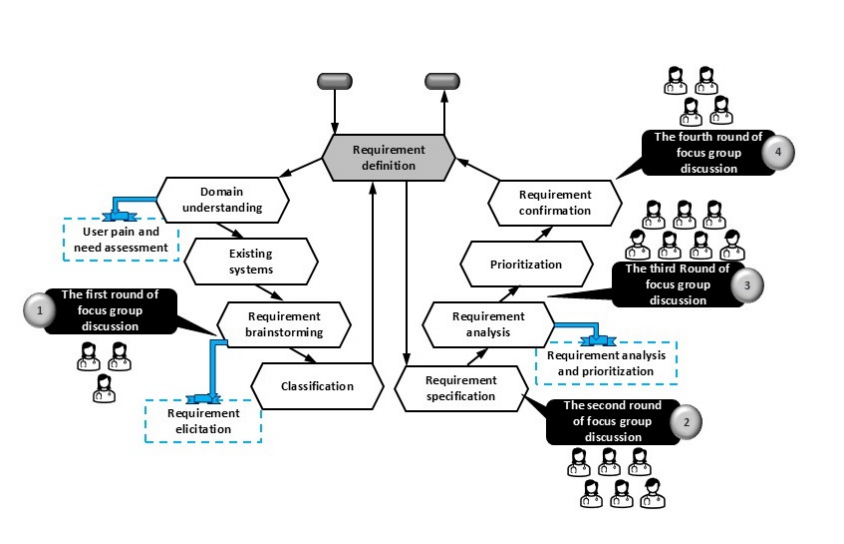
The process of system requirement definitions for data-driven medical software for electroencephalography seizure monitoring in the digital care pathway for epilepsy.

A 4-round Delphi study, widely used in health care research to gather and refine expert opinions, was conducted using focus group discussions with health care professionals from May to December 2023. The aim was to identify the system requirements for integrating data-driven medical software for EEG seizure monitoring in DCPE. Several steps were taken while conducting the Delphi study to define the system requirements, leading us to form a process, as shown in Figure 1. The Delphi approach was selected for its ability to collect expert opinions and refine the system requirements iteratively and systematically. Each round had distinct objectives as follows:

Round 1 of the Delphi study was dedicated to identifying the current issues in the DCPE and user needs. The focus was on integrating data-driven medical software for EEG seizure monitoring, emphasizing the user-centric approach. This round was designed to ensure that the user needs were at the forefront of the study, making the audience feel that their needs were being prioritized.

Round 2 aimed at defining system requirements based on expert feedback from the first round.

Round 3 of the Delphi study was a pivotal stage where the system requirements were prioritized and refined through expert discussions. This thorough process was designed to ensure that the system aligned with clinical needs, instilling confidence in the audience about its alignment with their requirements.

Round 4 confirmed and validated the system requirements, incorporating final expert feedback for development.

The requirement analysis process began with the initial definition of system requirements, where user needs and pain points were assessed. On the basis of the domain understanding and comparison of the existing system, a guideline was prepared and presented to the health care professionals in the first round of the Delphi study. With their extensive experience and knowledge, these professionals were instrumental in starting the requirement elicitation. The idea of data-driven medical software for EEG seizure monitoring integration in DCPE was presented, and requirement brainstorming was started. The main point of this round of the Delphi, according to the health care professionals, was as follows:

The main issue is when the patients are alone during their seizure, or no one is around them to monitor their seizure. It would be nice if we can have the track of the seizures using EEG as well as the video and recording of the seizure to check the symptoms, the type of the seizure.

Experts 1 and 2

Therefore, the user pain point is the lack of monitoring and assistance for patients during their seizures, especially when no one is around them. The user needs a system that can track and record the patient’s EEG and a video of the seizure to assess symptoms and seizure type and reduce the risk of any dangerous incident during the seizure. Currently, the hospital has video EEG recording systems in Finland that can track different types of seizures for several days up to 2 weeks. Monitoring and analyzing patients’ historical seizure data can help predict and prevent future incidents. On the basis of reviewing existing systems and the first round of Delphi study discussions, the monitoring systems for epilepsy were classified into five main categories based on their aims: (1) self-management and medication adherence, (2) EEG-based systems for seizure detection, (3) cardiac-based systems, (4) non-EEG seizure detection systems using wearables, and (5) video-tracking systems. The system requirements were updated based on the study in the second category, EEG-based systems for seizure detection.

After updating the system requirement, the second round of the Delphi study was started with the aim of a requirement specification. Six health care professionals attended the session for 60 minutes. The main points of this session were as follows:

The type of sensors used for seizure remote monitoring are challenging. We can start with commercially available devices that have already been validated. The system can be specified for Continuous EEG monitoring at the ICU first. It would be nice to add more electrodes with additional channels into the current validated devices.

Experts 1 and 2

Subsequently, SRA and prioritization were initiated, and the third round of the Delphi study was conducted. Seven health care professionals, including an EEG signal processing specialist, worked closely with the industry to create EEG sensors. This session lasted 55 minutes. The experts discussed the preferences and system requirement prioritization in this session:

Probably, it is good to have some preferences considering how the proposed system fits our plans. For example, jumping right into the home remote monitoring and putting much effort into it might be not easy. Still, we can bind the project around developing the University Hospital care pathway for status epilepticus. EEG monitoring for the patients with status epilepticus or sudden unconsciousness due to unknown etiology at the emergency department, neurological department, or intensive care unit, could be the first target of the system, especially for the neurological department or intensive care unit.

Expert 1

The commercially available device at the current form requires a server that is installed inside the hospital network. But since we already have this kind of server in use in the intensive care unit.

Expert 2

It is better to start with the adults at the emergency and ICU department. After the system is validated, it can be specialized for patients of different age groups, preferably above 12 years old. The system can be used for patients with different ages and demographics.

Experts 1, 2, and 3

On the basis of the discussion, the development of data-driven medical software for EEG seizure monitoring needs to be started for the emergency department at the hospital targeting adult patients. If the system is adopted successfully, it can be extended to a home monitoring system, and a video recording system can be added as an additional feature. The home monitoring system is part of a plan that requires a requirements analysis study and co-design sessions with patients as the primary users of the system, along with other stakeholders.

Compatibility with existing hospital systems, such as electronic health records and EEG monitoring infrastructure, was a primary consideration. The EEG-EpiDigi system is designed to integrate with current systems without requiring extensive modifications. However, the hospital is preparing the new infrastructure to accommodate the modern version of EEG-EpiDigi.

The final interview round was done in December 2023 with 4 experts to summarize and confirm the system requirements and the discussion of the previous rounds. The interview lasted around 55 minutes.

### Study Design

**Figure 2:**
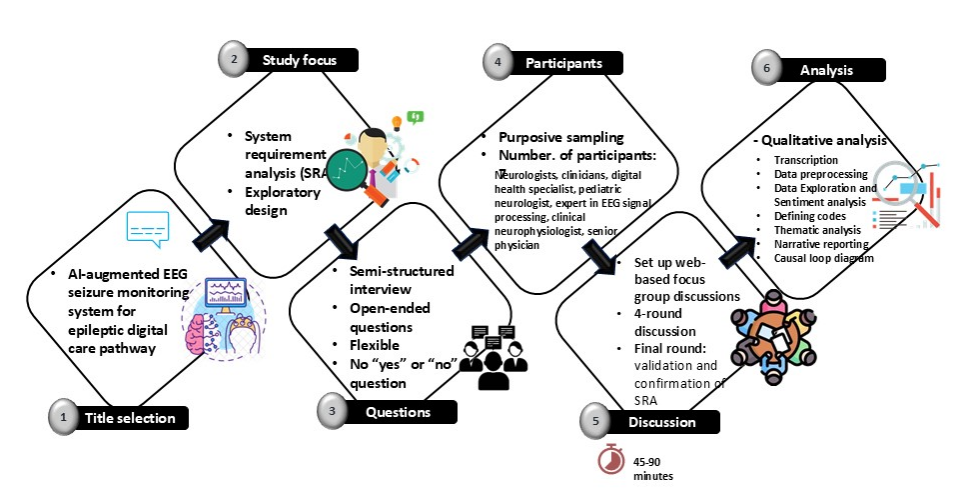
The study design for a 4-round Delphi study using focus group discussions. AI: artificial intelligence; EEG: electroencephalography; SRA: system requirement analysis.

To address the research questions, a 4-round Delphi study using focus group discussions was chosen to systematically gather expert opinions and iteratively refine the system requirements for data-driven medical software designed for EEG seizure monitoring (EEG-EpiDigi) in the DCPE (Figure 2).

The first round concentrated on identifying current issues within the DCPE and understanding user needs, focusing on integrating data-driven medical software for EEG-based seizure monitoring. In the second round, we aimed to define the system requirements based on the feedback received from experts in the first round. The third round prioritized and refined these requirements through further expert discussions to ensure the system met clinical needs. Finally, the fourth round confirmed and validated the system requirements, incorporating additional expert feedback to guide development.

After reviewing the existing systems and assessing the user needs and the shortcomings of the current DCPE based on discussion with health care professionals, data-driven medical software for EEG seizure monitoring was proposed, and the initial requirements were defined. The qualitative Delphi study using a focus group was centered on SRA for the proposed EEG-EpiDigi using exploratory design. A semistructured interview was conducted using a flexible, open-ended questionnaire. Each round of the Delphi study used a focus group that lasted 45 to 90 minutes and included 3 to 7 participants. The initial round of the Delphi study was conducted face-to-face, while the subsequent 3 rounds took place on the web via Teams (Microsoft Corp). This mixed method approach allowed for greater flexibility in expert participation, although it had a minimal impact on data collection and analysis. Thematic consistency was maintained across both formats, and the online discussions generated richer data due to the ease of participation. The first round focused on identifying user needs and the current issues related to DCPE. In contrast, the second to fourth rounds concentrated on defining system requirements, prioritizing them, and confirming their validity. The system requirements process was guided by the latest advancements in the field [17-19] and industry standards, including IEC 62304 for medical device software [20,21] and ISO 14971 for risk management [22-24]. These standards ensured that our approach aligns with best practices in medical software development. We analyzed the collected data qualitatively, coding the results to extract and present themes narratively, followed by interpretation.

### Participants

Purposive sampling aligns the sample with the goals and objectives of the study, thereby enhancing its rigor and the reliability of the data and findings [25]. For this study, purposive sampling was chosen to ensure that the expert panel is representative and diverse concerning epilepsy care and EEG monitoring. A group of health care professionals from Oulu University Hospital were selected, including neurologists, digital health specialists, pediatric neurologists, experts in EEG signal processing from the University of Oulu and industry, clinical neurophysiologists, and senior physicians. This diverse composition allowed for a comprehensive coverage of clinical, technical, and research perspectives. According to Burch et al [26], focus group discussions can be conducted with 4 to 8 participants for a minimum of 45 minutes. In this study, each round of the Delphi study was designed to include 4 to 7 participants and lasted between 45 and 90 minutes.

### Interview Guideline

**Figure 3:**
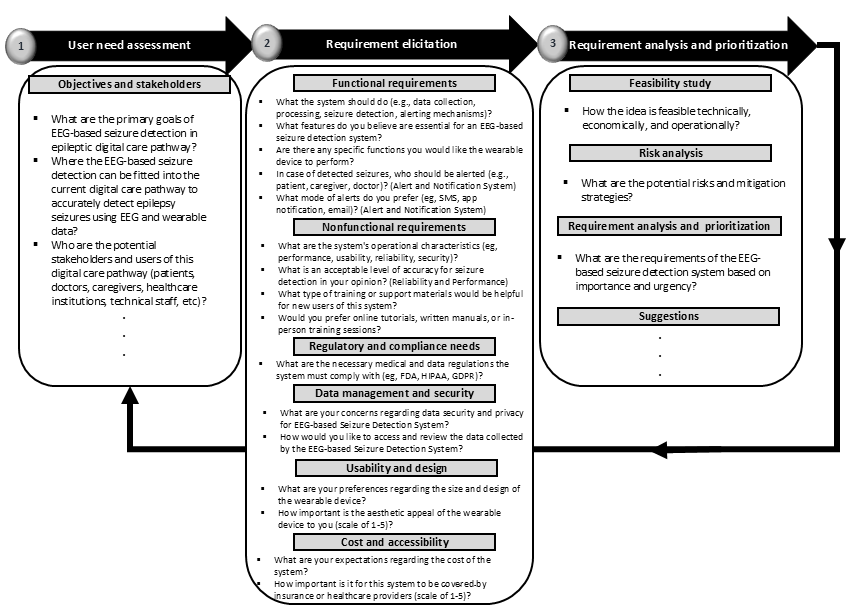
The interview guideline for a semistructured Delphi study using focus groups for requirement confirmation. EEG: electroencephalography; FDA: Food and Drug Administration; GDPR: General Data Protection Regulation; HIPAA: Health Insurance Portability and Accountability Act.

The Delphi study used focus group discussion and aimed at SRA for data-driven medical software for EEG seizure monitoring in DCPE. It was conducted using a semistructured interview guideline, as shown in Figure 2. To provide proper orientation, the interviewer was partially engaged in the discussion management. According to the studies by Keikhosrokiani [17], Catanio [27], Keikhosrokiani [28], and Grady [29], the interview guideline followed an iterative process from user need assessment to requirement elicitation, analysis, and prioritization. The iterative process encompassed 10 main topics: objectives and stakeholders, functional requirements, nonfunctional requirements, regulatory and compliance needs, data management and security, usability and design, cost and accessibility, feasibility study, risk analysis, analysis, and prioritization. The iterative process started from the initial round of Delphi and was enhanced for the next rounds. The interview questions were carefully designed based on a thorough review of relevant literature and insights from an expert panel, ensuring they aligned with the research questions. A pilot study was conducted before the primary research to improve the validity and clarity of the questionnaire. Feedback from this pilot study was essential in refining the questions, ensuring they effectively addressed the research objectives while remaining clear and accessible to participants. This iterative process significantly enhanced the reliability and relevance of the data collected from the Delphi study. Figure 3 illustrates the final version of the interview guideline, which was used for the fourth round of Delphi study discussions for requirement confirmation, composed in the previous rounds.

### Data Analysis

The data analysis steps for the focus group discussion are shown in the final step of Figure 1. The transcribed data were initially read before the data analysis process to ensure the data’s general content and scope. The data analysis began with data preprocessing and data cleaning to remove unnecessary words, symbols, etc, and the data were organized based on the topics. Next, data exploration using visualizations and sentiment analysis was performed to overview the story of the data at a glance. Afterward, data coding was done using qualitative tools to examine patterns, keywords, and concepts throughout the text. The broader themes representing larger text sections were defined based on the initial codes. The themes were reviewed to ensure they were coherent, distinct, and effectively represented the dataset. After explaining each theme, articulating what each cover was and how they relate to the content was done. Finally, the results were reported narratively with interpretation and quotations. A causal loop diagram was created for further analysis and discussion to complement the results.

### Ethical Considerations

Ethical approval from the regional medical research ethics committee of the Wellbeing Services County of North Ostrobothnia was granted for the EpiDigi project to involve human participants who are health care professionals and patients (approval number EETTMK: 59/2023). Verbal informed consent was taken from the participants. The names of the participants, products, and the company were anonymized. No compensation was provided to the participants.

## Results

### Data Exploration

**Figure 4:**
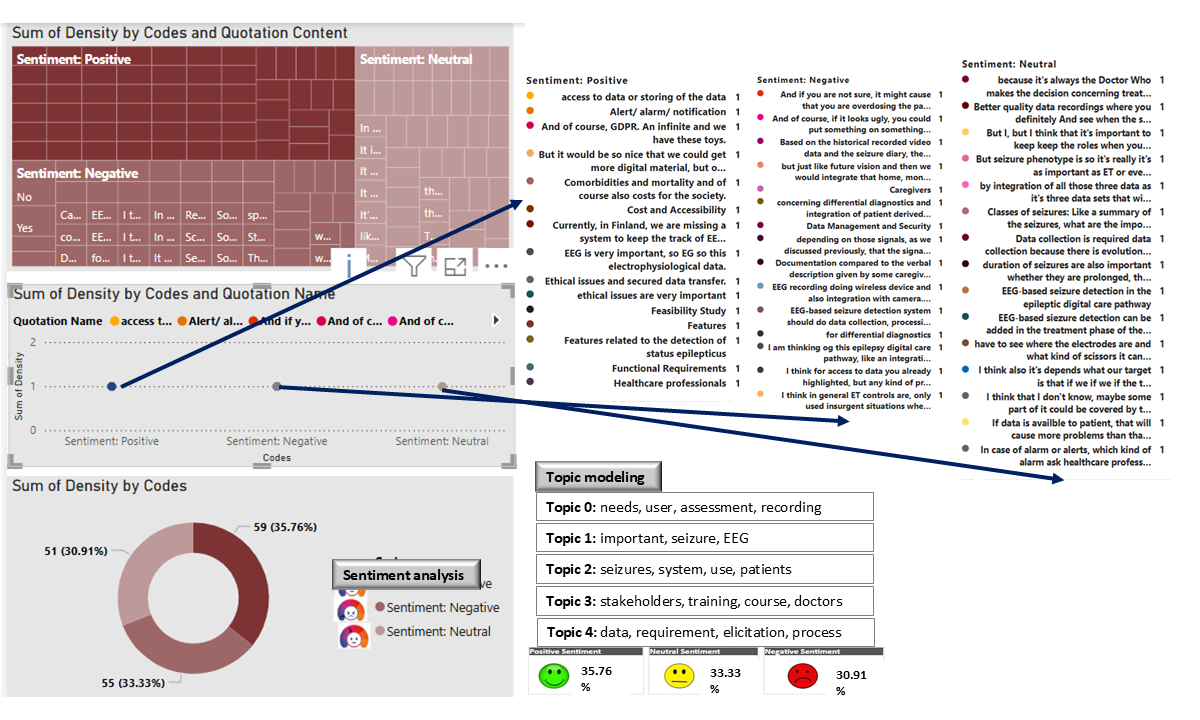
Data exploration for the interview using word frequency, topic modeling, and sentiment analysis. EEG: electroencephalography.

Data were explored from the interview transcripts before the thematic analysis to understand the overall data. Word frequency analysis, topic modeling, and sentiment analysis were used for text exploration, as shown in Figure 4.

The most frequent words in the word cloud indicated that health care professionals preferred wearable video EEG seizure detection systems at the hospital to provide accurate and timely data on seizure types for epilepsy classification and treatment decision-making. They requested courses to train them in using the proposed system and were interested in expanding the system for home monitoring using wearable devices.

On the basis of the topic modeling results, 5 main topics were highlighted from the data. Topic 0 focused on user needs and assessment, whereas topic 1 centered around data, seizures, EEG, and related concepts. Topic 2 discussed seizures, systems, use, and patients, while topic 3 concerned stakeholders, their views, and course of action. Finally, topic 4 was related to data requirements and elicitation processes.

Sentiment analysis was also performed on the data collected from the interviews. As shown in Figure 4, most sentiments toward the data-driven medical software for EEG seizure monitoring were positive, followed by neutral, and negative, respectively.

### Study Mind Map for the Qualitative Data Analysis

**Figure 5:**
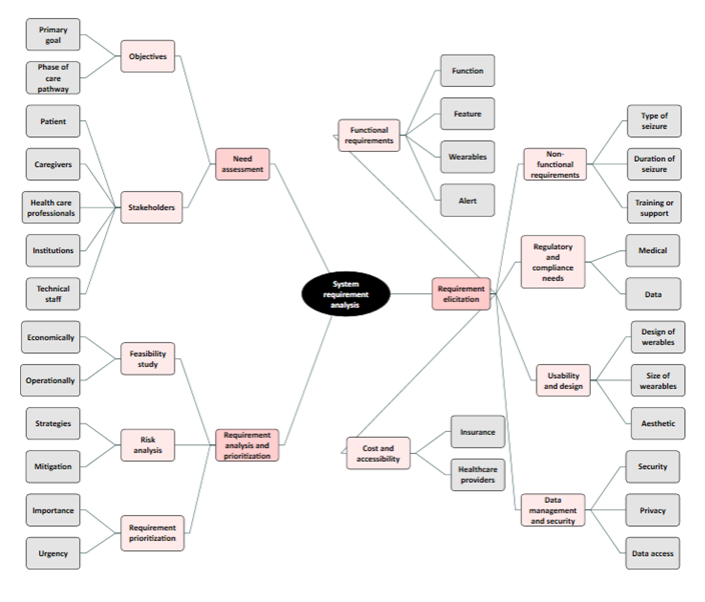
The study mind map for system requirement analysis.

The study mind map was prepared based on transcribed data to be used as a guide for SRA. As shown in Figure 5, SRA consists of 3 steps: user needs assessment, requirements elicitation, and requirements analysis and prioritization. The needs assessment evaluated the system’s aims, objectives, and stakeholders. In contrast, functional requirements, nonfunctional requirements, regulatory and compliance needs, usability design, data management, and security were reviewed in the requirements elicitation and feasibility study, and risk analysis and requirement prioritization were considered in the requirements analysis and prioritization step. The main keywords for each subevaluation are addressed in Figure 5.

As outlined in Table 1, SRA consisted of 3 steps: user needs assessment, requirements elicitation, and requirements analysis and prioritization. The needs assessment step evaluated the system’s aims, objectives, and stakeholders. In contrast, functional requirements, nonfunctional requirements, regulatory and compliance needs, usability design, data management, and security were reviewed in the requirements elicitation and feasibility study, and risk analysis and requirement prioritization were considered in the requirements analysis and prioritization step. The main keywords for each subevaluation are addressed in Table 1.

**Table 1:** The study mind map for system requirement analysis.

System requirements and subrequirements			Items
**No 1. Need assessment**			
	Objectives	Primary goals; the phase of the care pathway	
	Stakeholders	Patients; caregivers; health care professionals; institutions; technical staff	
**No 2. Requirement elicitation**			
	Functional requirements	Function; feature; wearables; alert	
	Nonfunctional requirements	Type of seizure; duration of seizure; training and support	
	Regulatory compliance and needs	Medical; data	
	Usability and design (Method section)	Design of wearables; size of wearables; aesthetic	
	Data management and security	Security; privacy; data access	
	Cost and accessibility	Insurance; health care providers	
**No 3. Requirement analysis and prioritization**			
	Feasibility study	Economical; operational	
	Risk analysis	Strategies; mitigation	
	Requirement prioritization	Importance; urgency	

### Users Need Assessment

#### Primary Goals

**Figure 6:**
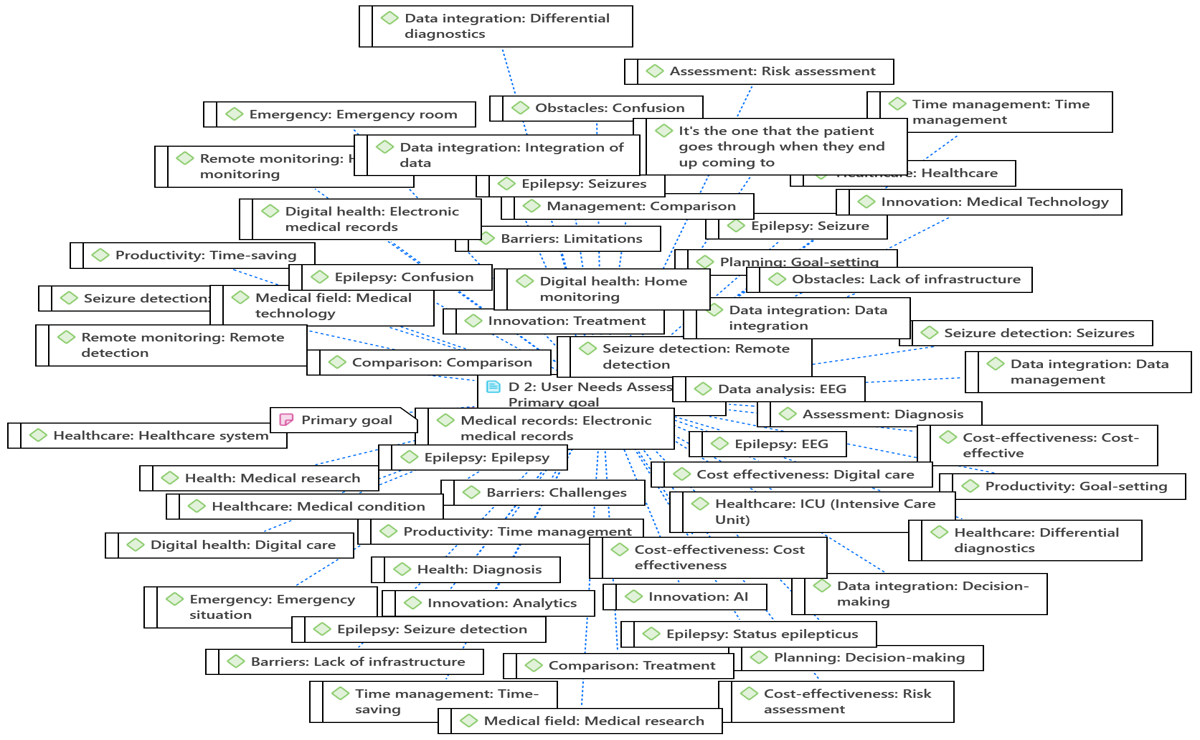
A sample network of codes for user needs assessment. AI: artificial intelligence; EEG: electroencephalography; ICU: intensive care unit.

After data were transcribed, coding was done to assist us in the thematic analysis. A coding network was generated for each part of the mind map, as shown in Figure 5. A sample of a network of coding for the primary goal of data-driven medical software for EEG seizure monitoring is illustrated in Figure 6.

The focus group discussion on user need assessment led us to some themes and subthemes, as summarized in Table 2. For instance, the experts mentioned the following:

EEG-based seizure detection is especially required in the emergency department for the status epilepticus phase. It is also helpful because we cannot get the 24/7 EEG recording from all those patients...

[W]e need continuous electrocardiogram (ECG) monitoring all the time in the ICU. We can proceed with adult patients first, then use it for children...

Thus, the primary goal was to implement EEG-based seizure detection specifically for emergencies, such as prolonged seizures or status epilepticus, starting from the emergency room to intensive care units (ICUs). A critical theme is the need to minimize *diagnosis time* during severe epileptic events, emphasizing rapid detection and treatment initiation. This discussion led us to the subtheme *enhancing emergency seizure management.*

Furthermore, the discussion emphasizes leveraging *remote detection technology to identify seizures*, potentially integrating AI-based analytics to enhance time and cost-efficiency. In addition, the discussion that “EEG-based wearable seizure detection system should be available and provided for doctors for differential diagnostics and treatment decision-making, especially if the seizure is prolonged...” and “Currently, we would need a recording, especially in the in the emergency in the status epilepticus phase” suggests the need for long-duration EEG recording, especially for status epilepticus.

Health care professionals continued the discussion by stating the following:

This wearable EEG-based seizure detection could help health care professionals in decision-making...thus, in this pilot process, we are focusing on emergency where you have a prolonged seizure, especially status epilepticus as a neurological emergency...We could start from the emergency room to the ICU intensive care unit.

EEG-based seizure detection is mentioned as a tool to aid health care professionals in deciding whether to admit patients to *intensive care,* especially in nonconvulsive status epilepticus or nonepileptic seizures. EEG-based seizure detection in the DCPE treatment is proposed to improve patient management.

Moreover, the experts highlighted that “there is a need for EEG analytical models to assist the neurologists in differential diagnostics, especially in the evenings and weekends...” It was argued that there is a need for the most suitable type of wireless devices and wearables for seizure recording and video systems to detect seizures, highlighting the importance of technology in monitoring and data storage. Predictive analytics based on historical recorded video data and seizure diaries can be used to predict future seizures and their types, indicating a need for advanced analytic tools in health care.

The experts further mentioned, “Since the EEG analysis is time-consuming, if we could utilize AI-based preliminary analytics before the decision-making of health care professionals, it would be very efficient and cost-effective.” This kind of solutions emphasizes the need for cost-effective solutions, suggesting that wireless EEG devices could aid in differential diagnostics and reduce the burden on clinical neurophysiologists. Furthermore, they emphasized the importance of integrating patient-derived data, device data, and electronic medical records for decision-making.

*Future integration* of home monitoring as part of the DCPE is touched upon as an extension of current hospital-based approaches.

**Table 2:** A Summary of themes, subthemes, and codes for user need assessment.

Themes and subthemes		Codes extracted from quotations
**Primary goal**		
	Enhancing emergency seizure management	Emergency, ICU^a^, intensive care, seizure detection, management, and effective productivity
	EEG^b^-based wearable seizure detection technology	EEG, seizure detection, and technology
	Health care decision-making and treatment	Treatment, decision-making, health care, and digital health
	Technological needs in monitoring and predictive analysis	Technology, seizure monitoring, remote monitoring, home monitoring, analytics, diagnosis, differential diagnostics, and predictive analytics
	Cost and resource efficiency	Cost-effectiveness and timesaving
	Future directions and needs	Limitations, challenges, barriers, medical field, and a lack of infrastructure
**Phases of digital care pathway for epilepsy**		
	Development of an updated digital care pathway for epilepsy	Planning, updated care, and emergency care
	Differentiation in seizure types	Differential diagnostics, seizure types, and status epilepticus
	Integration of wearable EEG–based detection	EEG, seizure detection, integration, and data integration
**Stakeholders**		
	Accessibility and use of medical data	Health care data, data analysis, and access
	Decision-making in health care	Decision-making, medical decision-making, health monitoring, treatment decisions, and expert opinion
	Data privacy and management	Confidentiality, patient privacy, patient engagement, patient data, and patient role
	Role of data in treatment planning	Data analysis, health care data, treatment planning, and disagreement

^a^ICU: intensive care unit.

^b^EEG: electroencephalography.

#### Phases of DCPE

As highlighted in the primary goals mentioned subsequently, the primary aim is to implement the proposed system for hospital environments first, specifically for the *treatment phase* on the current DCPE:

Developing a system for hospital environments and then proceeding with remote monitoring systems from home...differentiating between epileptic and non-epileptic seizures...plans to incorporate EEG recordings into the DCPE, focusing on emergency and treatment phases for patients, especially for those with drug-resistant epilepsy, can assist health care professionals...

If the patients successfully adopt the system, expanding it to the home remote monitoring system using suitably validated wearables is recommended.

The system’s initial development within a hospital environment would be feasible, emphasizing hospitals’ foundational role in the care pathway system. It mentions the subsequent *plan* to proceed with a remote monitoring system from the home, indicating a phased approach to implementation and an expansion of care beyond the hospital setting.

The importance of *epileptic versus nonepileptic seizures* was stated in the discussion to point out the need to separate seizures into epileptic and nonepileptic categories, suggesting a focus on specificity in diagnosis and treatment within the DCPE.

Plans to incorporate *EEG-based detection* into the DCPE were discussed, indicating a direction toward more advanced, technology-integrated solutions for monitoring and diagnosis. It was added that EEG-based detection is primarily used in emergencies and for follow-up treatment effects, but it is specifically aimed at health care professionals rather than patients. This highlights a focus on enhancing professional decision-making.

The pathway discusses a phased approach, starting with hospital-based systems and expanding to home monitoring. This indicates a comprehensive care strategy that extends beyond immediate hospital care.

#### Stakeholders

This section presents the user needs assessment focusing on various stakeholders, particularly relating to epilepsy management and the *use of data* in DCPE. The stakeholders identified include patients, caregivers, technical staff, institutions, and health care professionals. The document discusses the use of EEG recording data, seizure recordings, and other types of medical data.

The discussion suggests that the analysis of EEG recording should not be shown to patients or informal caregivers because it might not be beneficial and could lead to more problems than benefits. The assertion is that patients are generally not involved in decision-making related to diagnostics and thus might not need direct access to these data. In contrast, health care professionals are seen as requiring access to these data to make informed decisions about diagnosis, treatment, and care. The data can be transported or analyzed by another physician from a different hospital, indicating a need for data sharing and interoperability among health care institutions.

The discussion emphasizes the health care providers’ role in making treatment decisions, often during inpatient consultations. It highlights the importance of keeping roles defined, with the physicians and patients or caregivers working together based on the information provided by the health care providers. While not explicitly mentioned, the concern over patient *access to data* and the need for health care professionals to share and analyze data suggest underlying themes *of data privacy, security, and effective data management* in health care. The discussion iterates that health care providers use data to inform treatment options and discuss these with patients and caregivers, depending on the situation. Data are central to *treatment planning* and the patient–care provider relationship. Furthermore, the following implications for each stakeholder were highlighted:

- For patients, improved understanding and communication about why specific data are not shared with them might be necessary.
- For health care providers, there is a need for practical tools and systems to manage, share, and analyze patient data securely and efficiently.
- For technical staff and institutions, ensuring technological solutions’ reliability, usability, and security for data recording and predictive analysis is crucial.

#### Themes Relationships

**Figure 7:**
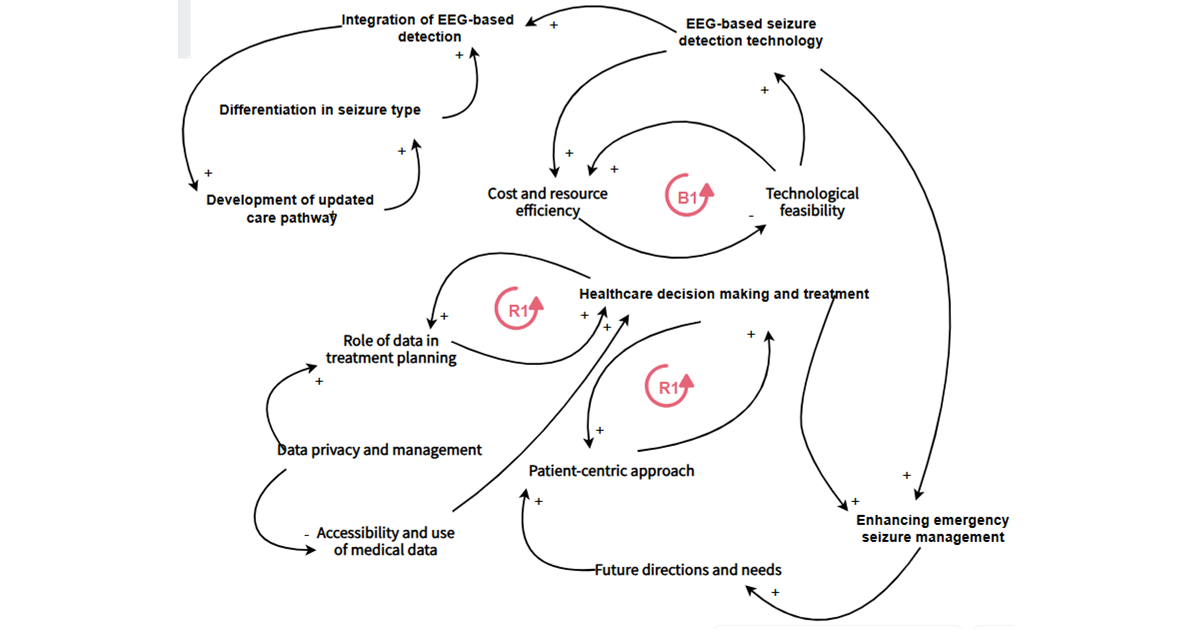
A causal loop diagram for the main objective. EEG: electroencephalography.

A causal relationship was created between the extracted themes, as shown in Figure 7, that shows the enhancement of emergency seizure management is significantly affected by the quality and accuracy of the EEG-based seizure detection technology. Furthermore, its improvement leads to more refined future directions and user needs, reflected in better patient-centric approaches. EEG-based seizure detection technology reduces the time and resources required for the system while improving cost and resource efficiency. Technological feasibility is the primary influence on the quality of EEG-based seizure detection technology. Better decision-making leads to improved patient outcomes, enhancing overall emergency seizure management. Improving cost-efficiency and resource efficiency enables further investment and improvement in the health care system. Balancing feedback might occur if costs reduce the ability to invest in newer technologies. Focusing on individual patient needs and outcomes (patient-centric approach) ensures the quality of emergency seizure management, and it is influenced by health care decision-making and treatment as better strategies and future technologies are developed.

As DCPE are updated and improved, there is a natural progression toward refining the understanding and categorization of conditions treated within those pathways, such as epilepsies. Once there is an established need or desire to differentiate seizure types more effectively, integrating more precise diagnostic tools such as EEG becomes a priority. With the integration of innovative technologies and diagnostic methods, the care pathway itself may be updated to incorporate these advancements, completing the loop and potentially starting a new cycle of updates and improvements.

A positive link between accessibility and the use of medical data and health care decision-making indicates that better accessibility improves decision-making capabilities. Moreover, informed decision-making positively influences how data are used in planning treatment. There is a negative link between data privacy and management and accessibility and the use of medical data, suggesting that inflexible data privacy policies might limit the accessibility of medical data. In contrast, adequate data privacy and management practices enhance the role of data in treatment planning. Finally, a positive feedback link between the role of data in treatment planning and health care decision-making illustrates that the effective use of data in treatment planning feeds back into improving decision-making in health care.

### Requirement Elicitation

#### Functional Requirements

**Figure 8:**
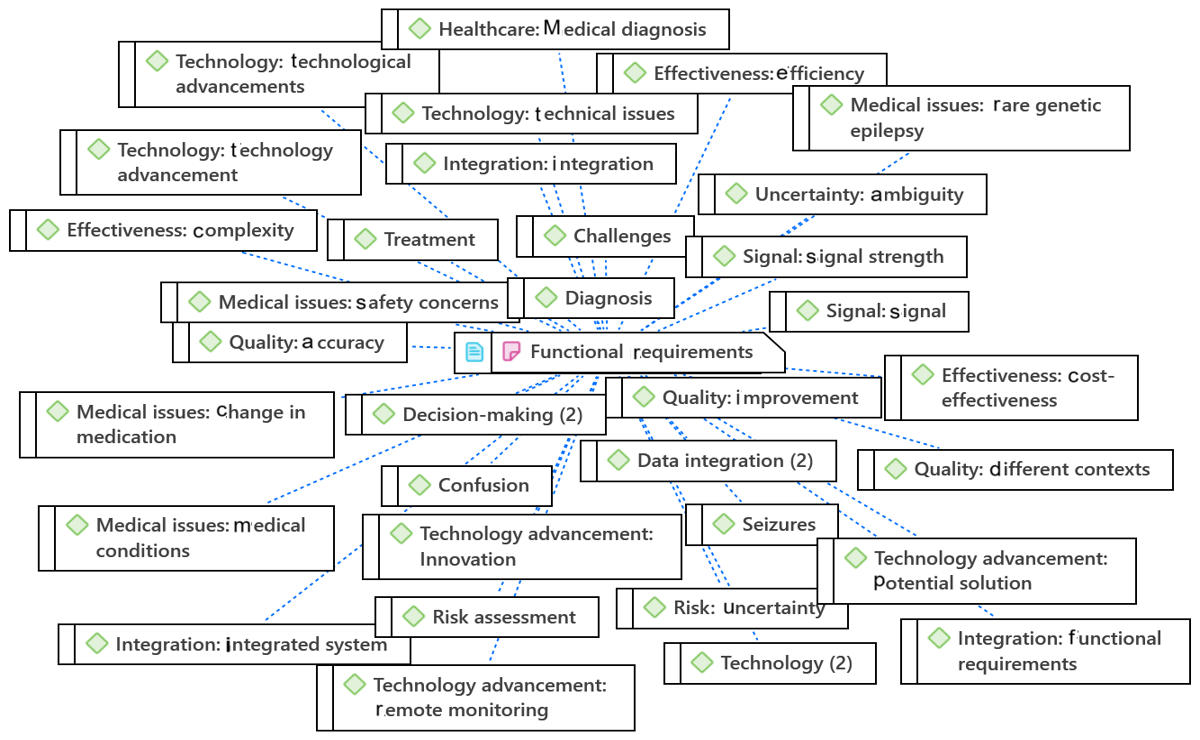
A sample network of codes for functional requirements.

On the basis of the focus group discussion with health care professionals, the coding network is shown in Figure 8, and the themes and subthemes are listed in Table 3—functional requirements emphasize the functions of the systems, features, wearables, and alert systems.

The conversation “the functional requirements are probably very different for home monitoring and hospital use...” notes the variation in functional requirements between home monitoring and hospital use, emphasizing the need for *contextual adaptable* solutions tailored to different settings. According to the discussion, “It would be good to integrate all types of data concerning the seizures in the system, such as motions, movement, sleep, video,” the system should *integrate various data types* (eg, motions, movements, sleep, and video) along with the number of seizures and device data to comprehensively understand the patient’s condition.

Efficiency in seizure detection, data processing, and *alerting* mechanisms was mentioned as a vital component. Furthermore, the challenge of differentiating between epileptic seizures and nonepileptic events was highlighted. The requirement emphasized the need for accurate diagnosis to prevent overtreatment and adverse events.

The evaluation of remote monitoring systems is closely linked to privacy and security risk concerns. Furthermore, real-time monitoring and the quality of service continue to pose challenges for remote monitoring services. Health care professionals identified several key issues with these systems, particularly concerning the types of wearable devices used for seizure detection, the accuracy of algorithms that classify seizures, and the overall trustworthiness of the system from the patient’s perspective.

One major challenge highlighted by health care professionals was the need for real-time access to patients’ seizure activity. Interviews conducted emphasized the evolving nature of epilepsy and stress the importance of continuous data collection and seizure phenotyping to adjust treatments and improve diagnostic accuracy. High-quality data recordings are crucial, especially for accurately capturing the onset of symptoms.

Integrating various data types, including video recordings, is essential for effective remote monitoring. The discussion also explored the potential of home remote monitoring systems that combine cameras, wireless EEG recording, and other technologies, envisioning a future where continuous and comprehensive monitoring is achievable. Wearable devices, which have already been tested, can be further refined for these remote monitoring systems.

The need for *user compliance*, especially in scenarios such as detecting prolonged seizures at night, was mentioned. The system should *effectively* alarm caregivers and integrate smoothly with hospital protocols. Furthermore, the discussion outlined various *features* or requirements for a system, possibly related to health care or patient monitoring, given the references to EEG, electrocardiogram, and ICU monitoring.

The discussion about *quality and usability concerns* started by highlighting the need for quality and ease of use in devices, particularly those related to brain monitoring (eg, EEG systems). This suggests a priority on user-friendly and high-quality *wearable devices* for monitoring purposes. There was a specific mention of using “good quality wearables and devices for monitoring.” This might refer to the need for reliable and accurate devices in a clinical setting, particularly for *continuous monitoring*.

The interview mentioned the need for continuous EEG monitoring in the ICU, indicating an emphasis on uninterrupted patient monitoring. This requirement is critical, starting with adult patients and extending to children. Moreover, the health care professionals discussed using *alarms* to detect nonconvulsive seizures. Emphasis on the placement of electrodes around the head and the reference to seizures spreading to the hemispheres suggested a focus on detailed and responsive monitoring systems that could detect and alarm medical staff about patient conditions. *Age-specific considerations were one of the critical points of discussion for the new system.*

The highlight of starting with adults and then extending monitoring capabilities to children indicates a phased, careful approach to implementing these technologies, likely due to the different physiological and anatomical considerations between adults and children. Prioritizing the features based on the *target demographics*, such as age group, and understanding which features are essential and desirable, requires stakeholder consultation.

For each requirement, technical feasibility and potential impact on patient care and health care workflow must be assessed [30]. Given the technical and clinical nature of the requirements, consulting with health care professionals and technology experts would be crucial in further refining and validating these needs. Additional steps would involve deeper stakeholder engagement, technical assessment of the proposed features, and a structured approach to integrating these requirements into a cohesive system design.

A health care professional stated the following:

We start with adults first, then we could implement a research project focusing on children...If we choose a device, it should be suitable for home use, minimize artifacts, and be user-friendly for patients or parents. It should be sensitive and specific...The device should inform users about seizures and be capable of detecting different kinds of seizures...We need to consider electrode placement and the types of seizures it can detect, focusing on devices that capture generalized seizures, particularly tonic-clonic seizures, while minimizing false alarms...Look into developing methods for ICU use, leveraging artificial intelligence...Determine the device’s suitability for children of certain ages.

**Table 3:** A summary of themes, subthemes, and codes for requirement elicitation.

Themes and subthemes		Codes extracted from quotations
**Functional requirements**		
	Contextual adaptability	Adaptable solution tailored to different settings
	Comprehensive data integration	Data type (eg, EEG^a^, motions, movements, sleep, and video)
	Alerting and detection efficiency	Alarm, alerting mechanism, and seizure detection
	Differential diagnostics	Differential diagnosis, epileptic seizures, and nonepileptic events
	Evolutionary data collection	Seizure phenotyping, treatment, diagnosis, and diagnostic precision
	Quality of data recording	Quality, data recording, capturing, and video recording
	Remote monitoring and technological integration	Home monitoring, remote monitoring, and wearables
	User compliance and system effectiveness	Prolonged seizures, night, alarm, and caregivers
	Quality and usability concerns	Ease of use, wearable device, user friendly, brain monitoring, and high-quality
	Use of wearables and devices	Good quality, wearable device, clinical setting, and continuous monitoring
	Continuous monitoring	Continuous EEG monitoring, uninterrupted patient monitoring, adult, and children
	Alarm for seizure detection	Health care professionals, alarms, responsive monitoring systems, and nonconvulsive seizures
	Age-specific considerations	Adults, children, age group, and features
	Target demographic	Differing physiological and developmental needs, adults, children, specific age, and design
	Usability and user friendly	Noninvasive, easy to use, user friendly, home care routines, simplicity, and intuitive design
	Technical specifications	Sensitivity, specificity, customization, and tonic-clonic seizures
	Information delivery	Inform, seizures, and communication
	Innovation and development	Innovative, artificial intelligence, and predictive analytics
**Nonfunctional requirements**		
	Types of seizures	Duration, nonepileptic, epileptic, nonconvulsive seizures, and tonic-clonic seizures
	EEG importance	EEG, vital, and seizure
	Seizure phenotypes	Types, variety, seizure, symptom, and abnormal
	Duration of seizures	Duration and seizure
	Materials and contents for training and support	Training, educational materials, on the web, in person, documentation, and digital content
**Regulatory and compliance needs and data management and security**		
	Access and storing of data	Complexity, sensitivity, handling patient data, storing, and access
	GDPR^b^ compliance	GDPR, regulations, and storage
	Ethical issues	Ethical considerations and authorized personnel
	Standardization and legal issues	Lack of standardization and regulatory frameworks
	Case study—Nelli system	Nelli system, developed by Tampere University and Tampere University Hospital
**Usability and design**		
	Size and aesthetic considerations for patient devices	Device size, patient movement, younger patients, customizable elements, colorful caps, children, less intimidating, user friendly, and hiding
	Designing for demographics and user-centered design in medical devices	Children, tailored design, personalized medical devices, patient’s age, user-centered design, and physical psychological needs
**Cost and accessibility**		
	Cost of implementation by hospital	Accessibility, affordable services, and brand-new hospital
	Cost of services by insurance	Insurance and services
	All public and private hospitals	Public and private
	Rural areas	Remote and rural areas

^a^EEG: electroencephalography.

^b^GDPR: General Data Protection Regulation.

On the basis of the aforementioned discussion, the themes related to wearables were extracted. The device was initially targeted at adults and then adapted for children. This indicates a phased approach, likely due to differing physiological and developmental needs. *Age specificity* is another crucial factor when selecting a device. The device’s suitability for children of certain ages is to be determined, indicating the need for customization or scalability in design. It was further discussed that the device should be *suitable for home use*, implying it needs to be noninvasive, *easy to use*, and potentially integrated with home care routines. It should be easy for the patients and parents to use, emphasizing simplicity and intuitive and *user-friendly* design. Regarding the *technical specifications*, the device must accurately detect seizures (high sensitivity) and not overreport nonseizure events (high specificity). It should minimize the recording of artifacts, which are irrelevant signals that could obscure seizure detection. Considering electrode placement and seizure types, it should be able to detect seizures, focusing on generalized and tonic-clonic seizures, and be amenable to customization or adaptable to various seizure types.

The *device should effectively inform users about seizures for information delivery*, indicating a need for clear alerts or communication mechanisms. The discussion about using current commercially available devices suggests leveraging existing innovative technology platforms as a starting point or benchmark. Considering the development of ICU use and leveraging AI, ambitions for advanced monitoring capabilities and predictive analytics are suggested.

#### Nonfunctional Requirements

The nonfunctional requirements were mainly emphasized in terms of seizure phenotypes, the importance of EEG, the duration of seizures, and training and support. While discussing the variety and importance of understanding different seizures, it was pointed out that although a seizure might be broadly categorized, the specific type and duration were critical. In addition, it emphasized the role of EEG in seizure detection and analysis, acknowledging its limitations since not all seizure types exhibited noticeable changes in EEG patterns depending on electrode placement. The significance of observable characteristics of seizures, such as facial expressions, eye movements, and other physical symptoms that are crucial for proper identification and need to be correlated with EEG data were highlighted. The importance of seizure duration, particularly in terms of the potential risks associated with prolonged episodes, such as status epilepticus was noted.

Regarding the types of seizures, the interview suggested a nuanced view of seizures, urging the need to understand different types and durations. For nonfunctional requirements, this theme emphasized the need for systems to accommodate a variety of seizure presentations and durations. EEG is recognized as a vital tool in seizure detection but is also noted for its limitations. Thus, there is a need for systems incorporating EEG data, but it is also possible to suggest integrating additional data sources and testing awareness. Moreover, the discussion described phenotypes as critical in seizure analysis, perhaps even more than EEG in some cases. This emphasizes the importance of comprehensive observational data in seizure monitoring software, possibly advocating for multimodal approaches. Duration of seizure was highlighted as a significant factor, especially concerning the risks associated with prolonged seizures. Systems must be sensitive to the duration of seizures, possibly incorporating alerts or interventions for protracted episodes.

The interview listed educational materials, including documentation, web-based tutorials, user manuals, in-person training sessions, and digital health parts. This theme explored the diversity and scope of materials available for epilepsy education and awareness. The materials were designed for different audiences, such as teenagers, caregivers, and patients.

A real-world application of EEG monitoring in a pediatric setting where a wearable device is used for seizure detection and continuous monitoring can be considered a practical example of this application. Incorporating case studies, such as a child with epilepsy who uses a wearable device that tracks seizures and alerts caregivers, could illustrate current technologies’ effectiveness and limitations. This example can clarify how the device recognizes seizure patterns and integrates with mobile apps for parents and health care providers.

However, some challenges remain unresolved in integrating data-driven software into the DCPE, such as data privacy and technical integration. Data privacy remains a critical issue in health care technology. A practical challenge is how an EEG device processes patient data while ensuring compliance with General Data Protection Regulation (GDPR). There is a potential risk of unauthorized access if role-based access is not strictly enforced, and the design must include secure data transmission methods, such as encrypted channels. For technical integration, exploring how the EEG device communicates with other medical systems could highlight potential barriers, such as interoperability issues, where devices from different manufacturers need help to share data effectively. Incorporating perspectives from various stakeholders, including health care professionals, patients, and regulatory bodies, can provide a more comprehensive view. For example, engaging physicians in discussions about the importance of EEG data alongside observable seizure characteristics can help identify what features are essential for them. Gathering feedback from patients and caregivers about their experiences with existing devices can emphasize the user-centered design approach. Moreover, including insights from regulatory bodies can shed light on the compliance challenges they face, addressing gaps in current frameworks for epilepsy monitoring technology. By integrating these elements, we can create a more robust discussion that addresses nonfunctional requirements and acknowledges the complexities of implementing health care technologies in a practical and user-friendly manner.

### Regulatory and Compliance Needs and Data Management and Security

On the basis of the interview discussion, the SRA revealed several key themes surrounding regulatory and compliance needs in health care, particularly regarding data management, GDPR compliance, ethical considerations, and specific case studies or systems.

The critical nature of where and how data are accessed and stored was mentioned in the discussion, especially in hospital systems and for research purposes, highlighting the complexity and sensitivity of handling patient data. In addition, GDPR is mentioned as an overarching and strict regulation affecting the storage and handling of data. The health care professionals suggested that GDPR compliance is a significant challenge that requires continuous discussion and resolution at the ministry level. Ethical considerations in accessing patient data were highlighted, emphasizing that only authorized personnel (eg, physicians and nurses involved in patient care) should access patient data. The importance of role-based access and strict data access logging to maintain privacy and accountability was underlined. The discussion further suggested a lack of standardization, particularly for patients with epilepsy, indicating a need for consistent legal and regulatory frameworks across different diagnostic groups. Finally, a specific system called the Nelli system at Tampere University was described [31]. It details a program for handling patient data, including AI scanning, anonymous analysis, and specialized handling by trained nurses or technicians. The system appears to be a part of a digital health care initiative, possibly relating to epilepsy treatment, as it includes familiarity with EEG techniques.

### Usability and Design

The “usability and design” discussion discusses the importance of size and aesthetics in medical devices, particularly those used by patients for home care or remote care in hospitals. On the basis of the extracted theme of *size and aesthetic considerations for patient devices, it was highlighted that* devices should be functional and stable without restricting patient movement, especially for younger patients. The devices should not cause discomfort or pain to the patient. Aesthetics can be improved by adding customizable elements such as colorful caps, particularly to appeal to children. The idea is to make the device less intimidating and more user friendly, hiding its medical nature.

To focus on designing for demographics and user-centered design in medical devices, making the devices less intimidating and more appealing to children implies a tailored design approach where the patient’s age and preferences are considered. This suggests a move toward more personalized medical devices catering to specific demographics. The emphasis on comfort, lack of restriction in movement, and aesthetic appeal point to a user-centered design approach. It reflects an understanding that medical devices should be designed to meet the end user’s physical and psychological needs beyond mere functionality. Human-centered design will be beneficial in the design of medical devices by reducing resources in software-related processes. It provides interactive and user-friendly solutions by focusing on user needs and requirements. User-centered design must involve software design, testing, and development phases

32

The discussion on size, aesthetics, and the balance between functionality and comfort in medical devices reflected broader themes in the medical device industry. These include the importance of user-centered design, the need for personalization in medical equipment, and the recognition that the success of a medical device is not solely dependent on its functional effectiveness, but also on its usability and acceptance by patients. Moreover, the health care providers mentioned the necessity of balancing technical requirements with human factors, advocating for designs that consider the patient’s physical comfort, psychological well-being, and overall acceptance of the medical device.

### Cost and Accessibility

Regarding the cost and accessibility of the proposed system, the interviewees mentioned that “maybe some part of the cost of the services provided by the new system can be covered by the patient or their insurance systems, but the costs should not be like prohibiting the use of the system...however, the hospitals can bear the cost of developing and implementing the system...the system needs to be accessible to all public and private hospitals...also in the rural areas...” Moreover, they mentioned, “The system would be essential and cost-effective for the whole society, and we cannot estimate the value at the individual level.” They concluded the interview by highlighting “Comorbidities and mortality and, of course, also costs for the society.”

### Requirement Analysis and Prioritization

**Figure 9:**
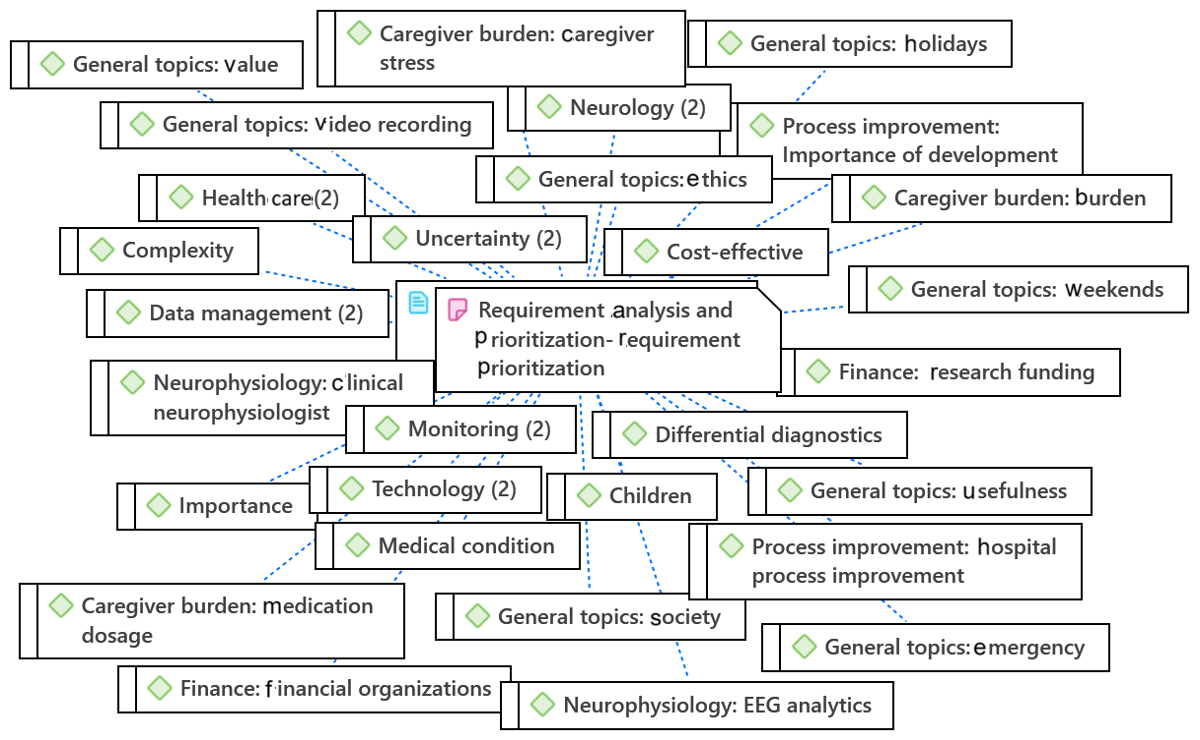
An example network of codes for prioritizing requirements. EEG: electroencephalography.

As discussed in the interview, some priorities were set for developing the EEG-EpiDigi. For instance, the system will be implemented in the hospital environment with a target on the emergency unit and ICU. The system will be used for adults first and expanded for other age groups. If the system is adopted successfully, remote home monitoring will be added as an extra module. The relevant themes were extracted based on the network of codes for requirement prioritization illustrated in Figure 9. The list of themes, subthemes, and codes extracted from the quotations for SRA and prioritization are shown in Table 3.

### Feasibility Study

In the focus group discussion, the health care professionals emphasized, “It would be more feasible to start from the hospital monitoring system and then expand it to a home remote monitoring system in the future.” Therefore, the following options must be considered to assess the feasibility of integrating data-driven medical software for EEG seizure monitoring into the DCPE. On the basis of the themes and subthemes outlined in Table 3, the *demand for hospital or home remote monitoring systems* needs to be carefully evaluated. This demand was assessed through expert feedback, focusing on current clinical needs and future potential for home monitoring systems. The idea of a home monitoring system is the next step after implementing it in the hospital. Therefore, data collection for requirement analysis and co-design sessions with patients will be performed after successfully implementing the proposed system in the ICU. The experts prioritized hospital-based monitoring (ICU and emergency departments) before expanding to home-based systems. Evaluation criteria included feasibility, scalability, and clinical relevance. Furthermore, *technological resources need to be carefully planned.* Before starting the project, the technology and infrastructure available for monitoring systems must be examined. In addition, the impact of monitoring systems on patient care and *health outcomes* needs to be determined. The financial implications of implementing and maintaining monitoring systems must be estimated. The feasibility of EEG-EpiDigi is high for hospital-based use, particularly in ICU settings. However, challenges include data privacy, integration with existing hospital infrastructure, and ensuring AI interpretability for clinical decision-making. Further research is required to address these challenges, especially for home-based monitoring.

The feasibility of the EEG-EpiDigi system, designed for managing epilepsy through digital tools, hinges on several critical factors outlined in Textbox 1, including technological integration, user acceptance, regulatory compliance, and data management.

**Figure 10:**
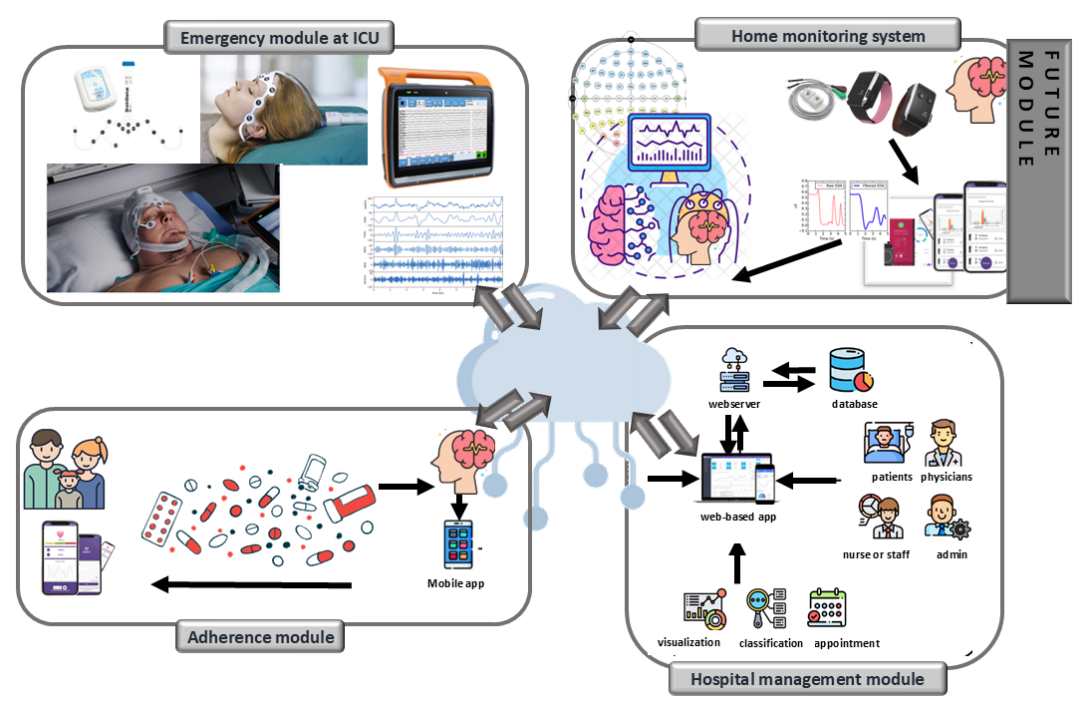
The proposed system architecture for EEG-EpiDigi in the digital care pathway for epilepsy. ICU: intensive care unit.

 Based on the study's results and assessing the feasibility, developing and integrating a system capable of monitoring EEG recording is required and feasible in DCPE (Figure 10).

**Key factors influencing the feasibility of the electroencephalography (EEG)-EpiDigi system.**

1. Technological integration: the successful implementation of EEG-EpiDigi will require seamless communication between EEG devices and other medical systems. Challenges include interoperability issues, where devices from different manufacturers need help to share data effectively. Ensuring that machine learning algorithms can accurately analyze the data from diverse sources is also vital. Creating standardized protocols and frameworks that enable this integration would be necessary while preserving data integrity.
2. User acceptance: engaging users, including patients, caregivers, and health care professionals, is crucial for the system’s success. The system must be designed with a user-centered approach, addressing the needs and preferences of different demographics. For instance, if the design does not consider the comfort and aesthetics of devices used by children or young adults, it may face resistance from these groups. Continuous feedback loops should be established to refine user experiences based on real-world inputs.
3. Regulatory compliance: navigating the complexities of regulations such as General Data Protection Regulation (GDPR) is a significant challenge. The system must ensure that all patient data are stored, accessed, and transmitted promptly. This includes implementing strict role-based access controls and maintaining comprehensive data access records. Moreover, as regulations evolve, the system may need to adapt to new compliance requirements, which could entail ongoing updates and modifications.
4. Data management and security: the EEG-EpiDigi system would need robust mechanisms for data management to handle sensitive patient information. Security features such as encrypted data transmission and secure storage are paramount to mitigate the risks of unauthorized access. In addition, ensuring the ethical use of data, including informed consent from patients, is necessary to build trust and acceptance among users.
5. Continuous monitoring and adaptation: the nature of machine learning systems requires ongoing training and validation of algorithms to ensure their effectiveness over time. This includes continuously updating the system based on new research findings and user feedback. The challenge lies in establishing systems for routine monitoring that can keep pace with the rapid advancements in AI and epilepsy research.
6. Stakeholder collaboration: collaboration across various stakeholders, including health care professionals, patients, and regulatory bodies, will be essential to address the multifaceted challenges of the EEG-EpiDigi system. Building a platform for dialogue can help ensure that the system meets clinical needs while adhering to compliance and ethical standards. In summary, while the EEG-EpiDigi system has the potential to revolutionize epilepsy management through enhanced data integration and user engagement, careful consideration of technological, regulatory, and user-centered challenges is necessary for successful practical applications. Engaging diverse stakeholders and implementing robust data management practices will be crucial for navigating these challenges and maximizing the system’s potential benefits.

### Risk Analysis

The risk analysis of the proposed system in DCPE depends on the treatment effectiveness, health care system coverage, diagnostic tools, and resources of health care professionals. This involves understanding treatment, management, and factors impacting their efficacy. *The health care system coverage* theme suggested what aspects of treatment or diagnosis are covered by health care systems. Finally, *diagnostic tools and indications* must be clarified to show how different diagnostic tools, such as EEG, are used and under what circumstances.

### Requirement Prioritization

The “requirement prioritization” discussion focused on prioritizing requirements for the data-driven medical software for EEG seizure monitoring for epilepsy, specifically in a hospital environment. On the basis of Table 4, the discussion emphasized the importance of a *step-by-step* approach to developing the system. Furthermore, it was mentioned that there is a specific focus on patients with *urgent neurological emergency* events, such as status epilepticus. *The* interview discussed the need for *EEG analytics* and video recording in hospitals, particularly during evenings and weekends, for differential diagnostics. *Children* were highlighted as a unique group for consideration due to their inability to make decisions or consent themselves. Moreover, the importance of data *storage and privacy*, especially in EEG-based systems, was mentioned.

The system is mainly aimed at helping patients with *drug-resistant seizures*. The system’s *utility during holidays* and weekends for private and government hospitals was noted, along with its voluntary nature. The discussion further underscored the importance of the system in terms of *cost-effectiveness* and its *value to society*. Moreover, it was highlighted that the implications of monitoring in chronic stages should be considered to avoid adding burdens on patients, families, and health care professionals. The discussion pointed out the need to carefully consider who the right person was to use the system on, which suggested the theme of decision-making *and responsibility*. The need to prioritize developing this system and securing funding from financial organizations was further emphasized. The system is intended to have a broad application across demographics for many users, including children, adults, teenagers, and older adults. The system must be successfully implemented for adults first, then expanded for other age groups. From this analysis, it is clear that the discussion was centered around developing and implementing a health care system with a strong focus on neurological emergencies, EEG analytics, and patient-centric considerations, especially regarding data privacy, accessibility, and cost-effectiveness.

**Table 4:** A summary of themes, subthemes, and codes for requirement analysis and prioritization.

Theme and subthemes		Codes extracted from quotations
**Feasibility**		
	Demand for monitoring systems	Hospital, home, and remote monitoring systems
	Technological resources	Technology, infrastructure, and start
	Health care outcomes	Patient care and health outcome
	Costs	Cost, implementation, maintenance, and implications
**Risk analysis**		
	Treatment effectiveness	Treatment, effective, and efficacy
	Health care system coverage	Aspects, treatment, and diagnosis
	Diagnostic tools and indications	Different diagnostic tools, EEG^a^, and circumstances
**Requirement analysis and prioritization**		
	Step-by-step process	Step-by-step, developing
	Focus on urgent neurological cases	Urgent, neurological, emergency events, and status epilepticus
	EEG analytics in hospitals	EEG, analytics, video recording, and differential diagnostics
	Special consideration for children	Children and special groups who are unable to make decisions give consent
	Data storing and privacy	Data, storing, and privacy
	Drug-resistant seizures	Drug-resistant seizures help patients
	Accessibility and utility during off hours	Holidays, weekends, and voluntary
	Cost-effectiveness and societal value	Cost-effective and society value
	Chronic stages monitoring	Chronic stages, burden, patients, families, and medical care
	Decision-making and responsibility	Careful decision, right person, and the use of the system
	Priority in development and funding	Secure funding, organizations, priority, hospital environment, and emergency
	Broad application across demographics	Wide range, different uses, children, adults, teenagers, and older people

^a^EEG: electroencephalography.

### Quality of Care

The paper outlines several strategies aimed at improving the quality of care and resource management within the context of the proposed EEG seizure monitoring system. The identified strategies and their development process are outlined in Textbox 2.

The strategies were developed through a systematic 4-round Delphi study involving focus group discussions with health care experts. Details on the 4 rounds are as follows:

1. Identifying current issues within the DCPE and gather user needs
2. Defining system requirements based on the initial feedback
3. Prioritizing and refining these requirements through further expert discussions
4. Confirming and validating the specific system requirements for development

This iterative approach ensures the strategies are well-informed and aligned with clinical realities and user demands.

While the paper does not provide specific case studies, the methods used (eg, focus group discussions and expert panels) are grounded in recognized best practices for medical software development. The emphasis on user-centered design and compatibility with existing technologies reflects practices successfully used in various health care settings to support implementing new systems.

Furthermore, the intention to start with adults in emergency and ICU settings is supported by existing literature highlighting the importance of targeted, evidence-backed implementations in enhancing patient outcomes and optimizing resource use. Future cases could arise once the EEG-EpiDigi system is operational, as real-world applications will provide concrete examples of how these strategies improve care quality and resource management.

**Figure 11:**
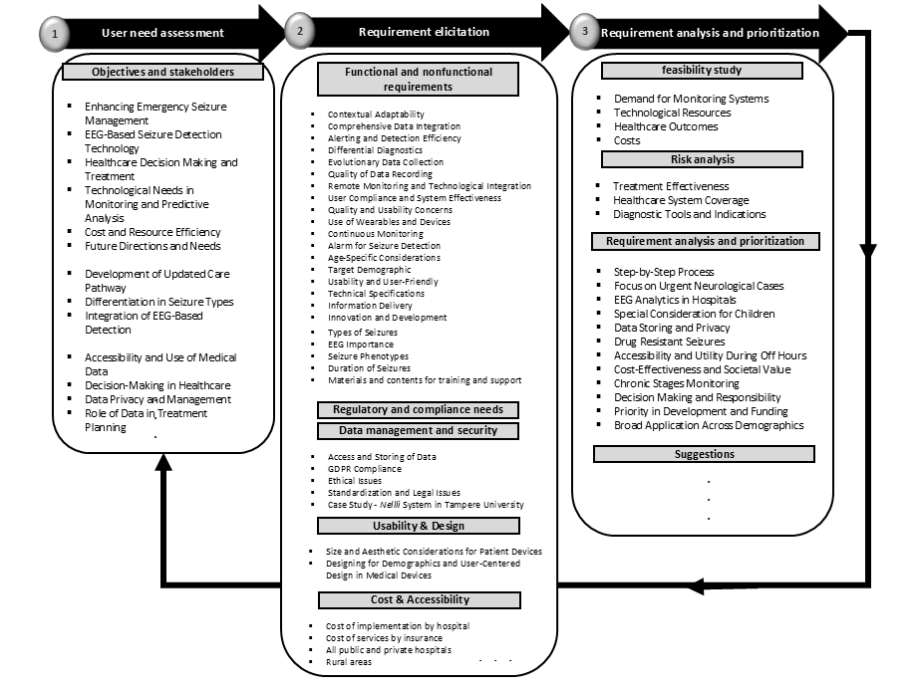
The proposed system requirement analysis (SRA-EpiDigi) for data-driven medical software for electroencephalography seizure monitoring in digital care pathway for epilepsy. EEG: electroencephalography; GDPR: General Data Protection Regulation.

 Based on the focus group discussion and thematic analysis results, this study proposed an SRA-EpiDigi for data-driven medical software to enhance EEG seizure monitoring quality in DCPE, as shown in Figure 11. SRA-EpiDigi addressed all research questions of this study.

**Strategies for improving care and resource management in electroencephalography (EEG) seizure monitoring.**

1. Integration with existing systems: ensuring compatibility with current hospital systems, such as electronic health records and EEG monitoring procedures, is vital for seamless implementation. This approach minimizes disruptions and enhances workflow efficiency.
2. User-centered design: the development of the EEG-EpiDigi system incorporates co-design sessions with primary users, including patients and health care professionals, to gather insights and preferences. This helps tailor the system to meet user needs, enhancing user satisfaction and care quality.
3. Phased implementation: focusing the system on adult patients in emergency and intensive care settings allows for more controlled and effective validation. Once the system proves effective, it can be expanded to cater to pediatric populations.
4. Focus on data-driven decision-making: using real-time EEG data aid clinicians in making informed decisions, improving responsiveness and treatment accuracy.
5. Feedback loops and iterative development: the Delphi study method ensures that expert feedback is continuously integrated into the design and development process. Each round serves to refine and adjust system requirements based on expert input.

## Discussion

### Principal Findings

The World Health Organization has stated that epilepsy is among chronic diseases causing the most comorbidity and mortality, and that it also incurs significant costs for society. It affects people of all ages, and it is one of the most common neurological diseases worldwide [2]. This highlights the severity and broader impact of epilepsy beyond the individual, affecting public health systems and society’s economic burden. As mentioned earlier, the health care professionals reviewed the current DCPE’s shortcomings in the focus group discussion. It was emphasized that despite several sophisticated electrocardiograms monitoring heart problems for decades, there are no comparable methods for EEG signal monitoring. The determination of seizure type is critical for choosing an appropriate medication, but predicting patient response to drugs is another challenge. About one-third of people with epilepsy have incomplete seizure control with current medicines, highlighting the need for more effective treatments [13]. Furthermore, the health care professionals highlighted the following:

Currently, the time taken to diagnose epilepsy is too long, and there is a need to minimize this duration. It would be a real risk to the patient, as the seizure could potentially prolong further.

Therefore, developing and integrating a system capable of monitoring EEG recording is required in DCPE (Figure 10). The focus group discussion outlines a need for advanced wearable wireless EEG–based seizure detection tools in emergency care for epilepsy, emphasizing quick diagnosis, efficient treatment, and cost-effective technology integration. However, implementing widespread sensor-based EEG seizure monitoring software, especially with AI integration, requires assessing technological feasibility and reliability. The solution would be cost-efficient, implying that budget and resource allocation will be key considerations. The patient-centric approach was suggested for further improvements in DCPE, especially in emergency contexts, pointing toward a more patient-centric approach focusing on rapid and personalized care. The study analysis revealed a comprehensive approach to enhancing care for epileptic seizures, suggesting significant implications for patient treatment, health care resource management, and technology integration in medical practices.

The proposed integration of EEG-based detection signifies an inclination toward the influence of technology to improve diagnostic accuracy and treatment effectiveness. This implies a broader digital transformation within health care systems and reflects an essential theme of enhancing professional capabilities while hinting at a need for more patient-centered tools in future developments.

The interview further outlines a focused, data-driven approach to developing a sustainable DCPE [32,33]. It prioritizes professional health care delivery, technological integration, and a phased expansion from hospital to home settings. Each theme reflects a commitment to improving care through specificity in diagnosis (differentiating seizure types), advanced monitoring technologies (EEG integration), and targeted user groups (health care professionals). The discussion suggests an evolving health care model that is adaptive, technologically advanced, and professional-centric. As health care continues to grow, such systems are crucial in guiding the development of more efficient, accurate, and comprehensive DCPE.

The functional requirements revolve around a versatile, accurate, and comprehensive seizure detection system that can adapt to different settings, integrate various data types, ensure high-quality recordings, and evolve with patient needs. There is an emphasis on balancing technological innovation with user needs and medical requirements to improve care for patients with epilepsy. Adopting and improving upon existing technologies and exploring data-driven systems, AI, and ICU applications show a commitment to cutting-edge solutions and broad applicability. The discussion, while brief, brings forward several essential aspects to consider in the context of nonfunctional requirements for seizure-related systems or analysis. It highlights the diversity and complexity of seizures, the roles and limitations of EEG, the importance of phenotypic observations, and the critical nature of seizure duration. Each of these themes has implications for the design and function of seizure monitoring, detection, and analysis systems, suggesting a need for comprehensive, adaptable, and sensitive approaches beyond traditional EEG data. This thematic analysis suggests directions for further research or development in enhancing seizure-related health care or monitoring technologies.

Furthermore, the discussion indicates the critical role of data in health care, the importance of clear roles and communication between patients and health care providers, and the potential of technology to improve patient care through advanced monitoring and predictive analytics. It also highlights the need to consider data accessibility and privacy concerns carefully. The study provides a glimpse into the complexities of health care regulation, especially concerning data handling, privacy, GDPR compliance, ethical considerations, and the need for specialized systems and training for health care professionals.

The study discusses the balance between making a functional and comfortable device for the patient. This includes not restricting patient movement, particularly for younger patients, and ensuring that the device does not cause discomfort or pain. Although functionality is paramount, aesthetics also plays a significant role in patient acceptance and comfort. The discussion suggests that aesthetics can be improved with customizable elements such as colorful caps, especially to make devices less intimidating for children. This shows a clear understanding that while the primary goal of a medical device is to serve a health-related function, its acceptance by patients, especially younger ones, can be significantly enhanced through thoughtful design that considers aesthetic appeal.

### Conclusions

In conclusion, this study incorporates health care professionals’ opinions, which can contribute significantly to a sustainable care pathway by providing insights and data-driven approaches that optimize resource allocation, improve patient outcomes, and integrate long-term solutions for patient care. Moreover, it offers valuable insights into the complex interplay among functionality, aesthetics, and user acceptance in medical system design. This research supports a thorough approach that considers patients’ physical and emotional needs, aiming to enhance the overall user experience and acceptance of medical software in various care settings. This study seems to be a concise set of suggestions (Figure 8) regarding improved health care technology for care pathways for epilepsy. It draws attention to the advancements in epilepsy health monitoring to highlight the gap and potential in neurological monitoring. It touches on public health themes, technology disparity, and the call for future-focused health care innovations.

The proposed SRA-EpiDigi for data-driven medical software for EEG seizure monitoring in DCPE requires ongoing improvements. One major limitation identified is the lack of patient involvement in the focus group discussions, which primarily included health care professionals. Since the system will initially be implemented within a hospital setting to aid health care providers in decision-making, it is essential to incorporate patient perspectives. To enhance the study’s future direction, emphasis should be placed on requirement analysis and co-design sessions, particularly for home monitoring systems tailored for people with epilepsy. The scope of development should not only include the EEG seizure monitoring software but also expand to real-time seizure video analytics.

In addition, these co-design sessions can effectively engage system developers and health care professionals in selecting optimal, user-friendly wearables and sensors for improved seizure management. This approach will ensure the system remains relevant and user friendly for health care providers and patients, thus enhancing its overall effectiveness and acceptance.

## Abbreviations

AI: artificial intelligence
DCPE: digital care pathway for epilepsy
EEG: electroencephalography
GDPR: General Data Protection Regulation
ICU: intensive care unit
SRA: system requirement analysis
