# Correction: Requirement Analysis for Data-Driven Electroencephalography Seizure Monitoring Software to Enhance Quality and Decision Making in Digital Care Pathways for Epilepsy: A Feasibility Study from the Perspectives of Health Care Professionals

**DOI:** 10.2196/79484

**Published:** 2025-11-11

**Authors:** Pantea Keikhosrokiani, Johanna Annunen, Jonna Komulainen-Ebrahim, Jukka Kortelainen, Mika Kallio, Päivi Vieira, Minna Isomursu, Johanna Uusimaa

**Affiliations:** 1 Empirical Software Engineering in Software Systems and Services Faculty of Information Technology and Electrical Engineering University of Oulu Oulu Finland; 2 Research Unit of Health Sciences and Technology Faculty of Medicine University of Oulu Oulu Finland; 3 University of Oulu Oulu, null Finland; 4 Oulu University Hospital Medical Research Center Neurocenter (Member of ERN EpiCARE) Oulu Finland; 5 Oulu University Hospital Oulu Finland; 6 Center for Machine Vision and Signal Analysis Faculty of Information Technology and Electrical Engineering University of Oulu Oulu Finland; 7 Cerenion Ltd Oulu Finland; 8 Research Unit of Clinical Medicine Faculty of Medicine University of Oulu Oulu Finland

In “Requirement Analysis for Data-Driven Electroencephalography Seizure Monitoring Software to Enhance Quality and Decision Making in Digital Care Pathways for Epilepsy: A Feasibility Study from the Perspectives of Health Care Professionals” [1], the authors made four changes to the authorship list.

First, Johanna Annunen and Jonna Komulainen-Ebrahim contributed equally, and have been credited as such with asterisks in the authorship list.

In addition, the second last and last authors, Minna Isomursu and Johanna Uusimaa, contributed equally as well. This has been added to the Acknowledgements section of the paper as follows:

MI and JU contributed equally to this work.

The affiliation of author JA has been changed from:

3. Oulu University Hospital, University of Oulu, Oulu, Finland

Author JA is now linked to the following affiliations:

3. University of Oulu, Oulu, Finland

4. Oulu University Hospital, Medical Research Center, Neurocenter (Member of ERN EpiCARE), Oulu, Finland

This has resulted in the renumeration of subsequent affiliations.

The affiliations of authors JA, JKE, MK, and PV have been changed from:

3. Oulu University Hospital, University of Oulu, Oulu, Finland

These authors are now linked to the following affiliations:

3. University of Oulu, Oulu, Finland

5. Oulu University Hospital, Oulu, Finland

This has also resulted in the renumeration of subsequent affiliations.

The correction will appear in the online version of the paper on the JMIR Publications website, together with the publication of this correction notice. Because this was made after submission to PubMed, PubMed Central, and other full-text repositories, the corrected article has also been resubmitted to those repositories.
